# Development and Validation of Artificial Intelligence Prediction of Epicardial Coronary Artery Spasm in Patients Without Obstructive Coronary Artery Disease

**DOI:** 10.3390/diagnostics16121847

**Published:** 2026-06-15

**Authors:** Ming-Jui Hung, Ian Y. Chen, Yung-Neng Lin, Nicholas G. Kounis, Patrick Hu, Chi-Tai Yeh, Claire Hung, Ming-Yow Hung

**Affiliations:** 1 Division of Cardiology, Department of Medicine, Chang Gung Memorial Hospital at Keelung, Chang Gung University College of Medicine, No. 222, Maijin Rd, Anle District, Keelung City 204201, Taiwan; miran888@ms61.hinet.net; 2 Division of Cardiovascular Medicine, Department of Medicine, Department of Radiology, Stanford Cardiovascular Institute, Stanford University School of Medicine, 300 Pasteur Drive, Stanford, CA 94305-5105, USA; iychen@stanford.edu; 3 Asia Vista Technology, 7 F., No. 755, Zhongzheng Rd., Zhonghe Dist., New Taipei City 23552, Taiwan; brian_lin@asiavista.com.tw; 4 Department of Cardiology, University of Patras Medical School, 26221 Patras, Greece; ngkounis@otenet.gr; 5 Department of Internal Medicine, School of Medicine, University of California, Riverside, CA 92521, USA; dr.hu.md@gmail.com; 6 Department of Cardiology, Riverside Medical Clinic, Riverside, CA 92506, USA; 7 Department of Medical Research and Education, Shuang Ho Hospital, Taipei Medical University, New Taipei City 23561, Taiwan; ctyeh@s.tmu.edu.tw; 8 Department of Medical Laboratory Science and Biotechnology, Yuanpei University of Medical Technology, 306, Yuanpei Street, Hsinchu City 30015, Taiwan; 9 Kang Chiao International School Xiugang Campus, No. 800, Huacheng Rd., Xindian Dist., New Taipei City 231049, Taiwan; s13435@kcis.com.tw; 10 Division of Cardiology, Department of Internal Medicine, Shuang Ho Hospital, Taipei Medical University, New Taipei City 23561, Taiwan; 11 Division of Cardiology, Department of Internal Medicine, School of Medicine, College of Medicine, Taipei Medical University, Taipei City 11031, Taiwan; 12 Taipei Heart Institute, Taipei Medical University, Taipei City 11031, Taiwan

**Keywords:** artificial intelligence, coronary artery spasm, risk prediction score

## Abstract

**Background:** Epicardial coronary artery spasm (CAS) is a frequent and important cause of myocardial ischemia. We aimed to develop and validate a noninvasive, artificial intelligence (AI)-driven risk score using routine clinical data to predict CAS in patients without obstructive coronary artery disease (CAD). **Methods:** This retrospective study analyzed a derivation cohort of 1050 patients and an external validation cohort of 600 patients who underwent intracoronary methylergonovine provocation testing between September 2008 and March 2025. A random forest (RF) model was developed using 15 clinical variables and simplified to a nine-variable model. Additionally, a convolutional neural network-long short-term memory (CNN-LSTM) deep learning model was implemented to predict CAS from raw digital electrocardiogram data (2611 electrocardiogram records). **Results:** The final nine-variable RF model, including predictors such as diastolic/systolic blood pressure, age, BSA, hemoglobin, smoking, heart rate, sex, and estimated glomerular filtration rate, demonstrated strong discriminatory power. The area under the curve was 85.8% (95% confidence interval [CI]: 85.8–89.9%) in the derivation cohort and 84.1% in the validation cohort (95% CI: 80.6–87.7%). A dose–response relationship was confirmed, with CAS prevalence increasing from 42.1% (0–1 risk factors) to 82.4% (≥5 risk factors). The electrocardiogram-based CNN-LSTM deep learning model achieved high sensitivity (91.4%) but limited specificity (11.9%); therefore, it should be considered a proof of concept rather than a clinical screening tool until further refinement is achieved. **Conclusions:** The nine-variable RF model provides a practical and accurate tool for early identification and risk stratification of CAS. The electrocardiogram deep learning model complements the RF model to improve clinical decisions and resource allocation in diagnosing CAS.

## 1. Introduction

Epicardial coronary artery spasm (CAS) is characterized by pronounced vasoconstriction of vascular smooth muscle cells, leading to total or partial narrowing of the lumen and potentially resulting in stable or unstable angina, myocardial ischemia or infarction, and sudden cardiac death [1,2,3]. With rising public awareness of CAS, cardiology decisions are more complex, requiring effective diagnosis and better patient care. While the Framingham Heart Study created the first formulas for predicting cardiovascular risk [4], most traditional coronary artery disease (CAD) models exclude unstructured data such as heart rate, which may oversimplify coronary health and overlook important predictive variables. Smoking, age, and C-reactive protein are risk factors for CAS, but contrary to CAD, CAS is not linked to traditional risk factors such as obesity, diabetes, and hypertension, indicating pathophysiological differences between the two conditions [5]. In contrast, while invasive provocative testing remains the gold standard for diagnosing CAS, noninvasive scoring systems can aid in earlier detection and guide treatment. Artificial intelligence (AI), incorporating computational algorithms to simulate and perform human problem solving and learning, is potentially useful in predicting cardiovascular outcomes, heart failure, arrhythmia and atherosclerotic/obstructive CAD. However, thus far, no AI-enabled algorithms and only one clinical score system for predicting CAS has achieved a high area under the curve of 0.952 [6], among which three score variables—namely, asthma, ST-segment elevation, and the hyperventilation test—warrant consideration. First, fewer than 4% of CAS cases involve asthma [7]. Second, initial electrocardiograms (ECGs) may be normal during early-stage or mild CAS [5]. Third, ST-segment depression occurs more commonly than elevation in CAS [5]. Fourth, the hyperventilation provocative test demonstrates reduced sensitivity in infrequent CAS attacks and may trigger multi-vessel CAS [5]. Consequently, the scarcity of alternative CAS risk prediction models underscores the importance of further research on CAS.

Because CAS plays a critical role in rapid plaque progression [8], and given the increased risk of myocardial infarction and all-cause mortality, accumulating evidence and recent professional society guidelines support early identification and treatment of CAS [9,10]. The complexity of ECGs and the varied symptoms and risk factors in CAS patients have presented significant challenges to the development of AI models. CAS risk is typically assessed using factors such as age, sex, medical history, smoking exposure, C-reactive protein levels, symptoms, and ischemic electrocardiographic changes during attacks [5]. Multiple risk factors may appear concurrently in an individual and can interact to elevate the likelihood of developing CAS [5], indicating that adopting a multifactorial strategy is the best for prevention of CAS. Timely detection and precise prediction of CAS play a vital role in reducing cardiovascular events, particularly among people with several minor abnormalities. Most CAS subjects have ≤4 risk factors, and coronary risk estimates are generally more accurate for those with fewer risk factors. Our study contributes to the field by developing and externally validating an interpretable AI-based model for predicting epicardial CAS in patients without obstructive CAD, an area that remains underrepresented in previous machine learning (ML) and deep learning (DL) studies. Unlike conventional CAD prediction models, our approach focuses on the distinct clinical profile of CAS and uses routinely available variables to support noninvasive risk stratification. The additional ECG-based convolutional neural network-long short-term memory (CNN-LSTM) model further explores the potential of DL to detect subtle ECG patterns associated with CAS. These contributions highlight the clinical relevance and novelty of our work in improving early identification of CAS and guiding the appropriate use of invasive provocation testing.

Although ML may improve cardiovascular risk prediction beyond conventional methods [11], its application in healthcare is hindered by methodological gaps in areas like algorithm blending, data standardization, feature optimization, and model tuning. Recent studies have applied ML and DL to coronary heart disease, particularly obstructive CAD, where models combining clinical variables, demographic factors, and coronary artery calcium scores have shown better discrimination than conventional pre-test probability scores, supporting the ability of AI to capture non-linear interactions among heterogeneous cardiovascular predictors [12]. Other studies have used routinely available noninvasive clinical parameters to develop ML-based CAD detection models, demonstrating that feature selection and algorithms such as support vector machines, logistic regression, random forests, and gradient boosting can provide clinically useful diagnostic performance [13]. In parallel, DL approaches have been applied to raw electrocardiographic data; for example, convolutional neural network-based models using resting 12-lead ECGs have shown feasibility for detecting CAD even when ECG abnormalities are subtle [14]. A recent systematic review further emphasized that ensemble ML methods are commonly effective for structured clinical data, whereas DL models are increasingly used for ECG and cardiovascular imaging data, although external validation, interpretability, and clinical workflow integration remain significant barriers to translation [15]. Despite these advances, most AI studies in coronary heart disease have focused on obstructive CAD, while CAS remains relatively underrepresented [16,17]. Over the past three decades, machine-learning methods—including support vector machines, boosted trees (such as adaptive boosting), and k-nearest neighbors—have advanced substantially. Since its introduction in 2001, random forest (RF) has been remarkably successful as a general-purpose algorithm for both classification and regression [18]. Across diverse benchmarking studies, RF consistently ranks among the most stable and best-performing methods, demonstrating strong predictive accuracy, robustness to noise, and minimal risk of overfitting [19]. Notably, RF also overwhelmingly outperforms traditional statistical approaches such as single decision trees, linear discriminant analysis, and logistic regression. For these reasons, we selected RF as the predictive modeling approach for this study. However, because CAS has distinct pathophysiological features, dynamic clinical manifestations, and diagnostic dependence on invasive provocation testing, there remains an important gap in the development of validated AI-based tools for noninvasive CAS prediction. Our study addresses this gap by developing and validating an interpretable random forest-based clinical prediction model, supplemented by an ECG-based CNN-LSTM proof-of-concept model, for identifying epicardial CAS in patients without obstructive CAD.

## 2. Material and Methods

### 2.1. Study Population

This retrospective study analyzed a prospectively enrolled patient cohort (September 2008–March 2025), all managed by Dr. Ming-Yow Hung at Shuang Ho Hospital in both clinic and hospital settings, ensuring complete data, consistent clinical decisions, and minimal treatment variability. During this period, 1050 consecutive individuals with suspected ischemic heart disease but without angiographic evidence of obstructive CAD underwent intracoronary methylergonovine provocation testing by coronary angiography within 2 months of angina. Patients were grouped by whether or not they had CAS. CAS was diagnosed by rest angina, reversible ST-segment changes relieved with sublingual nitroglycerin, and a positive intracoronary methylergonovine test. The comparison group consisted of patients with atypical, non–exertion-related chest pain and negative provocation test results. The exclusion criteria were obstructive CAD, prior coronary angioplasty or myocardial infarction, coronary microvascular spasm, severe valvular heart disease, inflammatory manifestations likely attributable to noncardiac conditions such as infections or autoimmune disorders, liver disease or renal failure defined as a serum creatinine level > 2.5 mg/dL, collagen disease, malignancy, and missing blood samples as part of the complete-case analysis. No patients demonstrated any allergic or hypersensitivity disorders. At Chang Gung Memorial Hospital, Keelung, Dr. Ming-Jui Hung managed another 600 consecutive patients who met the same enrollment criteria during this period. The larger hospital cohort was designated as the derivation cohort, whereas the smaller cohort served as the external validation cohort. Limiting each hospital to one attending physician ensured consistent decisions and reduced treatment variability in the study. The study was reviewed and approved by the Taipei Medical University-Joint Institutional Review Board (approval number: 201011004; date of approval: 20 December 2010) and Chang Gung Memorial Hospital Institutional Review Board (approval number 103-4592B; date of approval: 17 September 2014). Patient consent was waived due to this study being a retrospective analysis that does not use patient images. All patient information has been delinked and anonymized.

### 2.2. Clinical Data

Patients were evaluated for the presence of cardiac risk factors, including age, sex, cigarette smoking status, diabetes mellitus, hypercholesterolemia, and hypertension. Current smoking was defined as a smoking history of at least 0.5 pack-years and the use of at least 1 cigarette within 3 weeks before catheterization. Diabetes mellitus was considered present in patients receiving dietary management and/or pharmacological treatment for diabetes. The average baseline self-measured home blood pressure was calculated from at least 12 seated systolic and diastolic readings taken over a minimum of 3 days, ideally across 7 days. Hypertension was defined as a blood pressure >130/80 mm Hg or current use of antihypertensive medication. Hypercholesterolemia was diagnosed when the serum total cholesterol level >200 mg/dL. All patients underwent echocardiography before coronary angiography, during which heart rates were recorded, and the examination was performed within 2 weeks of the most recent angina episode.

### 2.3. Laboratory Analysis

On admission, data were collected for serum creatinine, estimated glomerular filtration rate, hemoglobin, hematocrit, platelet count, white blood cell/monocyte count, blood glucose, hemoglobin A1c, total cholesterol, triglycerides, high-density lipoprotein cholesterol, and low-density lipoprotein cholesterol. The estimated glomerular filtration rate was calculated from the Japanese equation: glomerular filtration rate (mL/min per 1.73 m^2^) = 194 × serum creatinine − 1.094 × age − 0.287 (if female, ×0.739) [20].

### 2.4. ECG Dataset Composition, Preprocessing, Model Architecture and Training Strategy

**Figure 1:**
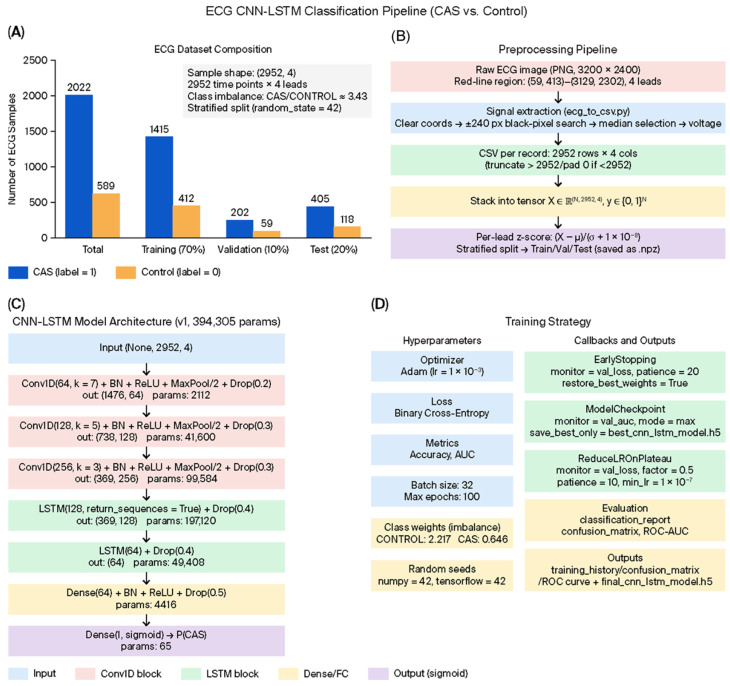
ECG CNN–LSTM classification pipeline for CAS versus control. (**A**) Dataset composition and stratified training, validation, and test split of CAS and control ECG samples. (**B**) Preprocessing workflow converting raw ECG images into fixed-length four-lead numerical signals, followed by tensor stacking and per-lead z-score normalization. (**C**) Hybrid CNN–LSTM architecture, in which Conv1D blocks extract local temporal ECG features and LSTM layers capture sequential dependencies before sigmoid classification. (**D**) Training strategy, including optimizer, loss, metrics, class weighting, callbacks, evaluation outputs, and saved model files. BN, Batch Normalization (normalizes layer inputs to stabilize and accelerate training); Conv1D, 1D Convolution (a one-dimensional convolutional layer used to extract features from time-series signals); CSV, Comma-Separated Values (a tabular data file format where values are separated by commas); ECG, electrocardiogram; ReLU, Rectified Linear Unit (a common activation function, f(x) = max(0, x)); Val, Validation.

We implemented CNN-LSTM, namely the ECG-AI model, to predict CAS from raw digital ECG data. The parsing criteria for ECG were based on ≤90 days before coronary angiography. The dataset included 2611 ECG records from Shuang Ho Hospital: 2022 in the CAS group (77.4%) and 589 controls (22.6%). Because multiple ECGs per patient were available, data were partitioned at the record level into training, validation, and test sets using a 70%/10%/20% split (1827/261/523 records), while maintaining the same class distribution across splits. Inputs consisted of synchronous 4-lead ECG signals with 2952 time points per lead (input shape: 2952 × 4) at standard gain (10 mm/mV). Signals were standardized using Z-score normalization (mean 0, standard deviation 1). To mitigate class imbalance during training, class weights were applied (control: 2.217; CAS: 0.646). Normalization parameters were stored for reproducibility (normalization_params_cnn_lstm.npz). A hybrid CNN–LSTM architecture (version v1) was implemented with 394,305 total parameters. The feature extraction module included three 1D convolutional blocks with kernel sizes of 7, 5, and 3 and filter sizes of 64, 128, and 256, respectively. Each block incorporated batch normalization, max pooling, and dropout (rate 0.30). Temporal dynamics were modeled using two stacked LSTM layers (128 units with return_sequences = True followed by 64 units), with dropout (rate 0.40) for regularization. The classification head consisted of a dense layer (64 units, ReLU), batch normalization, dropout (rate 0.50), and a sigmoid output node for binary classification. The model was trained using the Adam optimizer (initial learning rate 0.001) with binary cross-entropy loss, using a batch size of 32 for up to 100 epochs with early stopping. Validation area under the curve (val_auc) guided learning-rate scheduling and model selection. ReduceLROnPlateau was configured with monitor = val_auc, patience = 5 epochs, factor = 0.5, and min_lr = 1 × 10^−6^. EarlyStopping monitored val_auc with patience = 20 epochs and restore_best_weights = True. The final model was saved as final_cnn_lstm_model_v1.h5, and the best checkpoint by validation AUC was saved as best_cnn_lstm_model.h5 (Figure 1).

Binary cross-entropy with sigmoid output and categorical cross-entropy with 2-unit softmax are mathematically equivalent for binary classification (softmax with K = 2 reduces to sigmoid). We trained a parallel model using categorical cross-entropy. As shown in Supplementary Table S1, the categorical cross-entropy variant produced more balanced precision/recall across the 2 classes (control F1 0.21 vs. 0.17 in binary cross-entropy) but a lower overall AUC (0.488 vs. 0.537). Loss function choice did not change the fundamental discriminative ability of the model, consistent with our broader observation that the bottleneck lies in the underlying inter-ictal ECG signal rather than in optimization details.

### 2.5. Predictors of Interest

The objective of this study was to evaluate the utility and accuracy of a risk score—constructed from routinely available clinical measurements—to identify patients with undiagnosed CAS in the absence of obstructive CAD. Accordingly, we focused on candidate predictors that are widely used in clinical practice and incur minimal cost. A total of 15 variables were considered: age, sex, smoking status, body surface area (BSA), heart rate, systolic blood pressure (SBP), diastolic blood pressure (DBP), diabetes, hypertension, left ventricular ejection fraction, hemoglobin, estimated glomerular filtration rate (eGFR), platelet count, total cholesterol, and white blood cell count. Among these variables, we selected representative predictors from pairs of highly correlated measurements to avoid redundancy. Specifically, body surface area was chosen over body mass index; eGFR over serum creatinine; hemoglobin over hematocrit; and white blood cell count over monocyte count. For lipid parameters, total cholesterol was used as the representative measure. The variables highlighted in Results 3.2 were the last predictors included in the model.

### 2.6. Coronary Angiography and Intracoronary Methylergonovine Testing

Coronary angiography was performed within 2 months of chest pain onset using the standard Judkins technique via either the femoral or radial approach. Nitrates and calcium antagonists were discontinued for ≥24 h before the procedure. Left ventricular ejection fraction was calculated using Simpson’s method. Selective left and right coronary angiography was conducted in multiple axial and hemiaxial projections. Obstructive CAD was defined as a ≥50% reduction in luminal diameter after administration of intracoronary nitroglycerin, 100 μg [21]. If no obstructive CAD was identified, intracoronary methylergonovine provocation testing was subsequently performed. Methylergonovine (Methergin^®^; Novartis, Basel, Switzerland) was administered in a stepwise manner at doses of 1, 5, 10, and 30 μg, first into the right and then into the left coronary artery. The provocation test for CAS was considered positive when there was a >70% reduction in luminal diameter compared with the diameter after intracoronary nitroglycerin administration, accompanied by angina and/or ST-segment depression or elevation [5]. Provocation testing was terminated with intracoronary nitroglycerin, 100–200 μg (Millisrol^®^; G. Pohl-Boskamp, Hohenlockstedt, Germany). Reversal of coronary artery narrowing further supported the diagnosis of CAS. Spontaneous CAS was defined as relief of >70% diameter stenosis following administration of intracoronary nitroglycerin, 100–200 μg.

### 2.7. RF Model

An ensemble of 500 trees was constructed using mtry = 3 and a minimum of 75 observations allowed in terminal nodes. At each split, a randomly selected subset of predictors was evaluated to identify the optimal partition that maximized class separation. Individual predicted probabilities were obtained by averaging the tree-specific predictions across the entire forest. Variable importance (VIMP) scores were then used to rank the relative prognostic contributions of each predictor.

**Figure 2:**
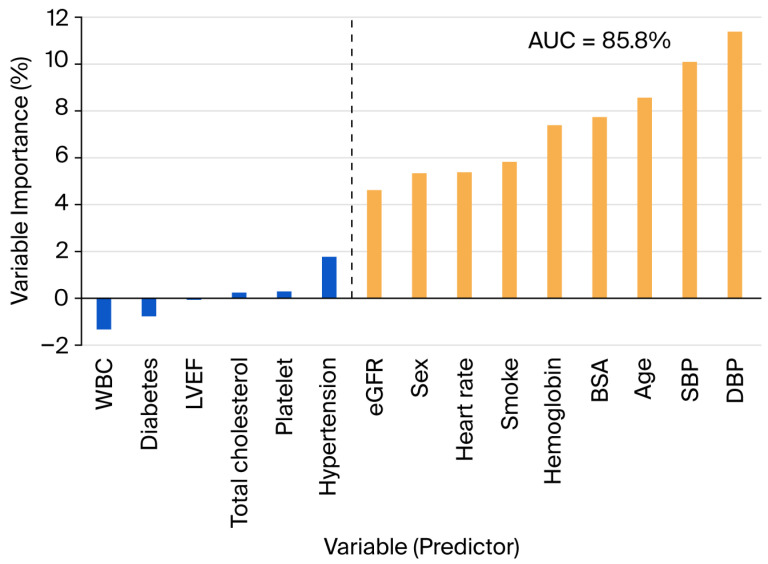
The relative importance of predictors included in the initial random forest model in the derivation cohort.

To select the final set of predictors in the RF model, we used VIMP rankings to identify the point at which the contribution of additional variables markedly diminished. In the initial RF model, predictors were ordered by VIMP from highest to lowest. When the VIMP values exhibited a pronounced drop at a particular rank (e.g., between the 9th and 10th variables out of 15), and the RF model constructed with the top 9 variables above that threshold already demonstrated strong discriminatory performance (e.g., AUC > 0.85), we selected that reduced set as the final model, thus constructing a 9-variable RF model (Figure 2).

### 2.8. Predictive Behavior Analysis of RF Variables

To examine how each of the 9 key predictors was associated with the risk of CAS, we generated partial dependence plots (PDPs) using established methodologies. These plots depict the nonlinear marginal effect of each individual predictor on outcome risk while holding all other variables constant. Among the 9 predictors, 2 were binary—current smoking and male sex—and both exhibited higher risk across the entire range of the PDPs. For the remaining 7 continuous predictors, we defined clinically meaningful thresholds based on both established clinical knowledge and the observed distributional patterns in the data. Specifically, risk factors were classified as DBP < 70 mmHg, SBP < 130 mmHg, age ≥ 60 years, body surface area between 1.7 and 2.0 m^2^, hemoglobin ≥ 13 g/dL, heart rate < 60 bpm, and eGFR between 90 and 120 mL/min/1.73 m^2^. These thresholds were selected by integrating the patterns observed in the PDPs with clinically grounded rationale.

### 2.9. Statistical Analysis

Comparisons of baseline demographic and clinical characteristics between groups (i.e., the derivation and validation cohorts; CAS vs. non-CAS control) were conducted using chi-square tests for categorical variables and independent samples *t*-tests for continuous variables. The 9-variable RF model was then applied to the independent validation cohort to assess external performance. To further examine the clinical relevance of the identified predictors, the entire study population was categorized into 5 groups according to the number of risk factors present (0–1, 2, 3, 4, and ≥5). The association between these strata and the likelihood of CAS was evaluated using univariable logistic regression, with individuals carrying no predictors (0–1 group) serving as the reference. In a separate analysis, the number of predictors was treated as an ordinal variable to assess the presence of a dose–response trend. RF model development and validation were performed in R version 4.4.1 using the “caret” package, while all remaining statistical analyses were conducted in SAS version 9.4 (SAS Institute, Cary, NC, USA). All tests were 2-tailed, and *p* < 0.05 was considered statistically significant.

## 3. Results

### 3.1. ECG Training Dynamics, Performance Evaluation and Confusion Matrix Analysis

The training process was halted after Epoch 31 by an automated early stopping process designed to prevent overfitting. The best model was identified at Epoch 11, where the training and validation metrics showed an optimal balance (validation AUC = 0.5436). Although later training cycles, such as Epoch 17, showed an increase in the validation AUC to 60.38%, this was accompanied by a significant drop in accuracy and higher loss, indicating overfitting of the training data. On a 523-sample test set, the model demonstrated a significant bias, achieving 73.42% accuracy but a low AUC of 0.5365. It excelled at identifying the CAS class with 91.4% sensitivity (370 true positives) but performed poorly on the control class, with only 11.9% specificity (14 true negatives). This imbalance resulted in a high false positive rate of 88.1%, as 104 control cases were misclassified as CAS, indicating the model tends to over-predict the CAS class (Table 1).

We re-evaluated the CNN-LSTM ECG-AI model using five-fold stratified cross-validation on the development set (*n* = 2088) and an independent hold-out test set (*n* = 523), with test-set predictions generated by ensembling the five-fold-specific models. The mean cross-validated AUC was 0.502 ± 0.033, and the ensemble AUC on the hold-out test set was 0.498 (Table 2). On the hold-out test set, the five-model ensemble achieved an accuracy of 0.7667, precision of 0.7813, recall of 0.9704, and F1 score of 0.8656; however, the AUC of 0.4976 indicated no meaningful discrimination beyond chance. These findings suggest that the apparent AUC of 0.5365 from the original 70/10/20 split was largely due to sampling variability in the hold-out test set rather than true discriminative performance.

### 3.2. Patient Characteristics

In the derivation cohort, 50% of participants were male and the mean age was 56 years, whereas the validation cohort included a higher proportion of men (58%) and had a slightly older mean age of 57 years (Table 3).

Compared to the derivation cohort, the validation cohort had more smokers, smaller body surface area, faster heart rates, higher blood pressure, greater prevalence of diabetes and hypertension, higher left ventricular ejection fraction, poorer renal function, less favorable lipid profiles, lower platelet and monocyte counts, and higher white blood cell counts. It is worth noting that the derivation cohort had a higher proportion of CAS (68%, 709 cases) compared to the validation cohort (48%, 289 cases). Due to significant variations in baseline characteristics and disease prevalence across the two cohorts, employing this independent validation cohort offers a robust and practical evaluation of the model’s external generalizability. The baseline characteristics of participants with and without CAS are presented in Table 4.

### 3.3. RF Model Development and Predictor Selection

An initial 15-predictor RF model, developed from a derivation cohort of 1050 patients (64% of 1650 total), achieved a strong AUC of 87.8% (95% CI, 85.8–89.9) (Figure 2).

BSA indicated body surface area; DBP, diastolic blood pressure; eGFR, estimated glomerular filtration rate; LVEF, left ventricular ejection fraction; SBP, systolic blood pressure; WBC, white blood cell.

VIMP analysis identified DBP as the most influential predictor (Table 5), followed by SBP, age, BSA, hemoglobin, smoking status, heart rate, sex, and eGFR. To assess model parsimony, we analyzed RF models utilizing varying numbers of predictors (Table 6).

**Figure 3:**
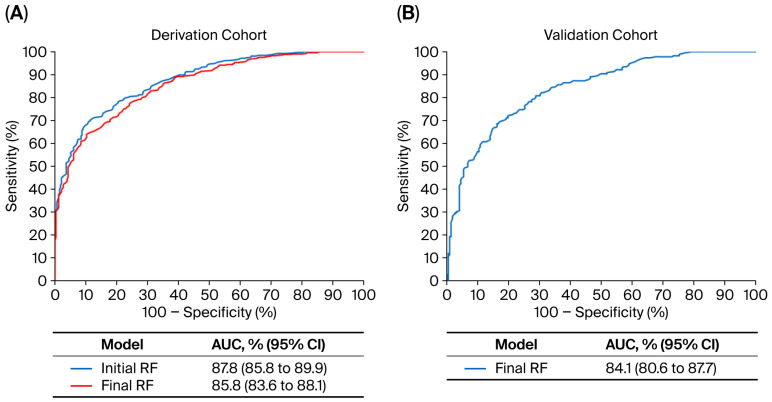
Discriminative performance of the initial and final random forest models in the derivation cohort (**A**) and performance of the final random forest model in the validation cohort (**B**). AUC, area under the curve; CI, confidence interval.

Based on these findings and a clear decline in VIMP after the ninth predictor, a more parsimonious nine-variable model was created, which demonstrated robust performance with an AUC of 85.8% (95% CI, 83.6–88.1) in the derivation set with a minor decrease of about 2% from the full model (Figure 3A). The nine-variable model’s robustness was confirmed through external validation on a cohort of 600 patients, yielding an AUC of 84.1% (95% CI, 80.6–87.7%) (Figure 3B). In our study, the performance of K-nearest neighbors and Support Vector Machine models was notably inferior, with AUC values of 70.6% and 79.9%, respectively. In addition to AUC, the nine-variable model showed similar accuracy in the derivation and validation datasets (76.3% and 75.8%, respectively), with a precision of 74.8% in both cohorts, a recall of 98.0% and 79.0%, and F1 scores of 84.9% and 76.9%, indicating strong sensitivity in the derivation cohort and balanced performance in the validation cohort.

**Figure 4:**
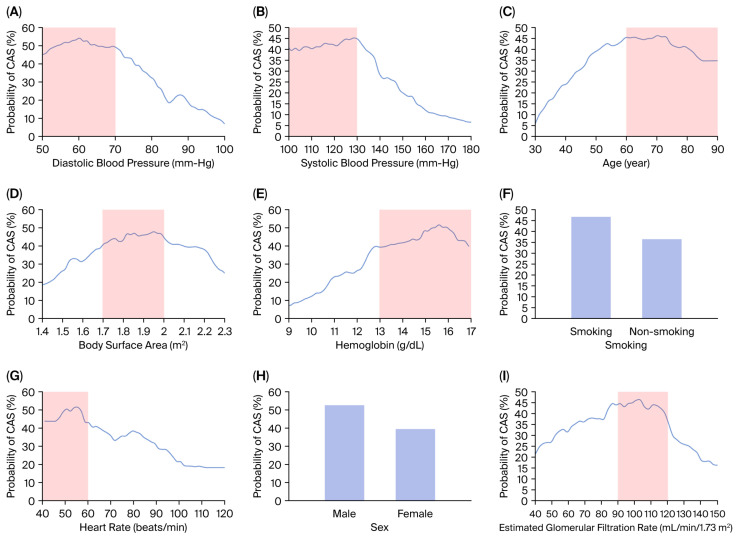
Partial dependence plots derived from the final random forest model in the derivation cohort, including diastolic blood pressure (**A**), systolic blood pressure (**B**), age (**C**), body surface area (**D**), hemoglobin (**E**), smoking (**F**), heart rate (**G**), sex (**H**) and estimated glomerular filtration rate (**I**). The light red–shaded area indicates the range along the *X*-axis where the probability of CAS is relatively higher. CAS, coronary artery spasm.

Partial dependence analyses were also conducted to illustrate the functional links between each predictor and CAS risk (Figure 4).

### 3.4. Prognostic Implication of Identified Risk Factors

**Figure 5:**
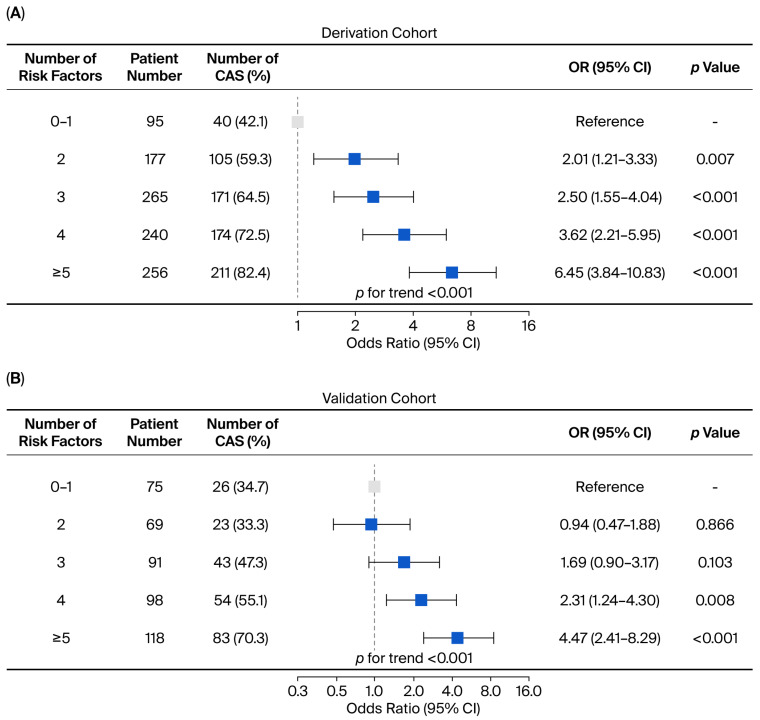
The probability of CAS stratified by the number of predictors in the (**A**) derivation and (**B**) validation cohorts. Variables based on the thresholds from the partial dependence plots were determined from the partial dependence plots. CAS, coronary artery spasm; CI, confidence interval; OR, odds ratio.

CAS risk assessment showed that event rates rose stepwise with more predictors, a trend seen in both derivation and validation cohorts (Figure 5).

In the derivation cohort, CAS prevalence increased with additional risk factors: 42.1% (0–1), 59.3% (2), 64.5% (3), 72.5% (4), and 82.4% (5 or more) (P for trend < 0.001; Figure 5A). A comparable monotonic gradient was observed in the validation cohort, with CAS rates of 34.7%, 33.3%, 47.3%, 55.1%, and 70.3% across the respective strata (P for trend < 0.001; Figure 5B). Notably, the validation cohort had different baseline characteristics and CAS prevalence compared to the derivation cohort, yet the consistent dose–response relationship in both groups highlights the model’s robustness and broad generalizability.

## 4. Discussion

The primary strength of the proposed ECG CNN-LSTM model lay in its high sensitivity (91% recall for CAS); however, this model requires significant improvement in specificity before it can be considered suitable for clinical screening. To enhance risk prediction using the RF algorithm across diverse populations, the derivation cohort (68% CAS prevalence) and validation cohort (48% CAS prevalence) demonstrated significant baseline differences, providing a robust test of external generalizability. VIMP analysis of the initial 15 candidate predictors identified DBP, SBP, age, BSA, hemoglobin, smoking status, heart rate, sex, and eGFR as key predictors. This streamlined nine-variable model achieved an AUC of 85.8% (95% CI, 83.6–88.1%), only marginally lower than the full 15-variable model (87.8%), and maintained strong external validity with an AUC of 84.1% (95% CI, 80.6–87.7%). A clear dose–response relationship between the number of risk factors and CAS prevalence was observed in both cohorts (derivation: 42.1–82.4%; validation: 34.7–70.3%; P for trend < 0.001). We demonstrated for the first time that a parsimonious nine-variable RF algorithm incorporating key hemodynamic and clinical predictors provides a robust and generalizable risk stratification model for CAS prediction across diverse populations.

Recent advancements in AI and DL have significantly enhanced ECG interpretation, revealing diagnostic details that surpass the capabilities of human interpreters. This is particularly evident in the context of CAD, where the diagnostic accuracy of conventional ECG analysis has long been recognized as limited. A study by Mahmoodzadeh et al. involving 400 patients with suspected CAD reported a sensitivity of 51.5% and specificity of 66.1% for 12-lead ECGs, with AUCs ranging from 0.524 to 0.586, underscoring the challenges of diagnosing CAD from a resting ECG [22]. A DL model by Sun et al. demonstrated strong performance in ruling out CAD, achieving an AUC of 0.81 and a high sensitivity of 93%, despite a low specificity of 30% [23]. With a 95% negative predictive value, it effectively rules out CAD and aligns with current guidelines [23]. Similarly, the ECG2CAD model developed by Kany et al. showed robust performance across large cohorts, with an AUC between 0.747 and 0.782 for detecting prevalent CAD [24]. Conversely, diagnosing CAS from a resting ECG is challenging due to the brief nature of spasm episodes. The poor control class metrics in Table 1 (precision = 0.29, recall = 0.12, F1 = 0.17) reflect three combined factors: (1) the 3.4:1 class imbalance (CAS:Control = 2022:589), which biases the classifier toward the majority class even with class weighting; (2) the limited information content of inter-ictal four-lead ECG signals for distinguishing CAS from healthy control—confirmed by five-fold cross-validation AUC of 0.502, categorical cross-entropy AUC of 0.488, and K-nearest neighbors AUC of 0.527 (Supplementary Table S2); and (3) record-level splitting allowing multiple ECGs per patient to be present in both training and test sets, which we acknowledge as a study limitation. Our model, while conceptually ambitious in its attempt to detect CAS from ECGs recorded up to 90 days before angiography, is an academic proof-of-concept not yet suitable for clinical use. Its low AUC indicates a weak signal, although its high sensitivity suggests it may be capturing a non-specific pattern associated with CAS. Future work will focus on methodological improvements, such as re-training and validating the model using strict patient-level splits for accurate performance estimates, to enhance the model’s performance and generalizability.

ML models, particularly RF, excel at predicting individual disease outcomes by identifying complex, non-linear patterns in diverse datasets that traditional statistical methods often miss, especially in fields like cardiovascular disease. RF is well-suited for clinical data, which is often small and noisy, as it uses ensemble learning to reduce overfitting and improve stability. Unlike other ML models such as k-nearest neighbors, support vector machines, or adaptive boosting, RF requires minimal preprocessing and is less sensitive to noise and class imbalance, making it a reliable choice for disease prediction research [25]. While ML has been effective in predicting cardiovascular disease, prior CAS prediction studies have relied on traditional risk scoring rather than modern ML methods [6,26,27]. leaving a gap in the literature. The three existing diagnostic scores for CAS have limitations, such as over-reliance on hyperventilation tests [6], applicability to limited patient populations in acute coronary syndrome patients without persistent ST-segment elevation [27] or limited to a single Taiwanese center with no external validation, raising concerns about generalizability to diverse populations and real-world clinical settings [26]. Furthermore, methodological weaknesses in some studies, such as the use of random train-validation data splits [6,27], may lead to overly optimistic performance estimates. In contrast, the RF algorithm is widely recognized as a leading AI algorithm for its balanced combination of accuracy, robustness, and interpretability in disease prediction [28].

Hypertension is less common in CAS-induced angina than in classic angina [29], and several studies have linked low blood pressure, particularly low DBP, to an increased risk of CAS [30,31,32]. This is supported by our findings that non-hypertensive smokers are at a greater risk for CAS [2]. In rats, hypertension reduces coronary artery contractile responses to serotonin [33], and contractile smooth muscle cells, not synthetic ones, are mainly involved in CAS development [34]. Mechanistically, low blood pressure, particularly low DBP, may reduce coronary perfusion and enhance endothelin-induced constriction, contributing to CAS [35]. The higher heart rates and blood pressures observed in our validation cohort may reflect different pathophysiological profiles. Although the exact mechanism of CAS is unclear, stimulation of both sympathetic and parasympathetic systems can induce CAS, suggesting a link to autonomic dysregulation [36]. Right CAS often leads to inferior wall ischemia, bradycardia, and supra-His conduction disorders, while left CAS can cause infra-His blocks. As a result, sinus bradycardia is more common than sinus tachycardia in CAS [37], supporting our finding that CAS is associated with a slower resting heart rate [36,37].

Age, smoking, and male sex are significant risk factors for CAS, with smoking having a stronger effect on younger individuals and men [38,39]. Smoking promotes CAS through various pathways, including inflammation, endothelial dysfunction, and increased blood viscosity [40,41]. However, a substantial portion of CAS patients are non-smokers (ranging from 25% to over 60% in some studies) [26,30,39], and there are notable differences in CAS prevalence between populations with similar smoking rates, suggesting the influence of other genetic or environmental factors [42]. Men are more prone to epicardial CAS than women [2,5], which may be partly due to a higher prevalence of smoking [43]. In cases of nonobstructive CAD, men are more likely to experience epicardial CAS, while women more frequently have microvascular CAS. Studies show that middle-aged women have higher resting coronary blood flow and smaller coronary arteries than men [44], likely due to differences in cardiac autonomic regulation [45], leading to a lower coronary flow reserve [46]. For a given level of coronary stenosis, women often exhibit a higher fractional flow reserve than men due to sex-based differences in vasomotion [47], despite similar microvascular resistance indices in both sexes with ischemia and nonobstructive CAD [46]. The higher prevalence of epicardial CAS in men than in women may be attributed to reduced resting coronary flow, lack of estradiol-induced vasodilation, lower fractional flow reserve, and higher smoking rates.

The variables were selected to minimize multicollinearity and redundancy among closely related measures while retaining clinically interpretable predictors. BSA is a more accurate indicator of metabolic mass than body mass index and is an independent predictor of coronary artery size and cardiovascular risk [48]. Higher BSA may lead to higher insulin secretion [49], which can lead to insulin resistance [50] and endothelial dysfunction, both of which are linked to CAS [51,52]. BSA was selected instead of body mass index because BSA is the widely used method for normalizing cardiovascular and echocardiographic measurements across different body sizes, whereas body mass index primarily reflects adiposity rather than cardiovascular size scaling; recent prognostic evidence also supports BSA-based indexing of cardiac and aortic measurements across body mass index categories [53]. Therefore, BSA is a valuable clinical variable for assessing coronary inflammation and related cardiac risks.

Hemoglobin was selected over hematocrit because hemoglobin directly reflects the blood’s oxygen-carrying capacity, whereas hematocrit represents the proportion of red blood cell volume and may be affected by plasma volume status or indirect calculation methods [54]. Furthermore, hemoglobin inhibits endothelium-dependent relaxation [55]. In patients with JAK2V617F myeloproliferative neoplasms, oxidative stress from erythrocyte-derived microvesicles increases the risk of myocardial infarction even without significant CAD [56]. Both low and high hemoglobin levels are associated with worse outcomes in CAD, following a J-shaped curve [57]. Conversely, mild anemia can be beneficial in cases of cerebral spasm by lowering blood viscosity [55]. In non-ST-segment elevation acute coronary syndrome, patients with CAS tend to have higher hemoglobin levels than those with obstructive CAD [27,58]. Our previous research indicated a dose-dependent effect of hemoglobin on CAS development in women but not in men [41], suggesting that hormonal differences may play a role [59,60].

eGFR was chosen over serum creatinine because eGFR provides a more clinically meaningful estimate of renal function, while serum creatinine is influenced by non-renal factors such as muscle mass, diet, and tubular secretion [61]. In patients with acute coronary syndrome, those with CAS tend to have a higher eGFR than those with obstructive CAD [27,58,62]. We previously demonstrated that non-diabetic CAS patients have higher glycated hemoglobin levels than non-diabetic non-CAS controls [63]. While CAS and coronary microvascular dysfunction are viewed as points along a continuum, recent studies suggest a link between high glycated hemoglobin, elevated eGFR and coronary microvascular dysfunction, indicating a connection between insulin resistance, glomerular hyperfiltration, and CAS [63,64]. The proposed hypothesis is that metabolic dysregulation, indicated by high glycated hemoglobin levels, contributes to glomerular hyperfiltration, coronary microvascular dysfunction and CAS. However, a low eGFR could also be a risk factor for CAS [65]. Further research is needed to clarify how these factors interact and which eGFR levels increase the risk of CAS compared to obstructive CAD. White blood cell count was chosen over monocyte count because total white blood cell count is a broader, routinely available marker of systemic inflammation and has been shown to be a strong and independent predictor of coronary risk, whereas monocyte count represents only one leukocyte subtype [66]. Finally, total cholesterol was used as the representative lipid parameter because it is a conventional component of lipid assessment and cardiovascular risk estimation, including established cardiovascular risk calculators, and provides a clinically familiar summary measure of lipid burden [67].

Unlike previous CAS prediction scores, which had limitations such as reliance on tests with moderate sensitivity or applicability restricted to acute coronary syndrome, this RF model uses widely available clinical data and offers external validation across populations with different baseline characteristics and CAS prevalence. However, this study had several limitations. First, the CNN-LSTM ECG model suffered from overfitting and class imbalance, resulting in low specificity and high false-positive rates. The five-fold cross-validation reported here employed record-level splitting; ECG records from the same patient may therefore appear in different folds. Although this remains a limitation, the cross-validated AUC of 0.502 ± 0.033 is already at chance level, indicating that further enforcing patient-level splits is unlikely to reveal additional discriminative ability. Patient-level GroupKFold validation is left to future multi-center work where larger sample sizes per patient are available. Second, despite the use of class weights to address data disparities, the AI-enabled CNN-LSTM hybrid architecture for the detection of CAS using ECG demonstrated a bias towards the majority CAS class. This is further highlighted by the low specificity of 11.9%, which implies a high rate of false alarms and could lead to unnecessary secondary examinations if the model were used as a standalone diagnostic tool. Reviewing alternative cutoffs (Youden-optimal, 0.3, 0.7) did not yield a clinically usable specificity profile, consistent with the chance-level AUC observed in cross-validation. Future work will focus on improving specificity through advanced data augmentation, incorporating focal loss, and exploring attention mechanisms to better balance the classification performance. Third, traditional methods assume linear relationships and often require strict handling of missing data, whereas ML can flexibly model complex, non-linear patterns with fewer restrictions [25]. Despite their capabilities, ML methods encounter similar issues with confounders and bias as traditional analyses. Selection bias can still affect how broadly the results apply, regardless of the method used. Misclassifying exposures or predictors due to self-reporting, poor measurement, or limited historical data can lower a model’s predictive accuracy, especially if errors are systematic. Fourth, this study used data from Asian centers, so its findings may not apply to other populations. Additional research with more diverse ethnic and racial groups is needed for validation. Finally, while having a single physician manage each cohort ensures consistency in diagnosis and treatment protocols, it does introduce potential bias related to individual practice patterns, emphasizing the need for future multicenter studies involving diverse practitioners to further validate the generalizability of our model.

## 5. Conclusions

We created two ML models for CAS risk prediction. A CNN-LSTM model using raw four-lead ECG signals showed potential for first-stage large-scale population screening with 73.4% accuracy and 91% recall, but its low specificity limits standalone use. To improve CAS risk prediction across diverse populations, an RF model was trained and validated on cohorts with significantly different baseline characteristics and CAS prevalence (68% and 48%), streamlining 15 candidates into nine key predictors: DBP, SBP, age, BSA, hemoglobin, smoking, heart rate, sex, and eGFR. This parsimonious model achieved an AUC of 85.8%, which was comparable to the complex 15-variable model (87.8%), with strong external validity (AUC 84.1%). A significant dose–response relationship between risk factors and CAS prevalence was identified in both cohorts, thereby validating the model’s utility for clinical risk stratification. This ML approach balances accuracy and interpretability, aiding clinicians in early CAS diagnosis and prevention while demonstrating the value of explainable AI in healthcare.

## Figures and Tables

**Table 1:** Classification report by class in the test set for the CNN + LSTM ECG-AI model.

Class	Precision	Recall	F1 Score	Support
Control	0.29	0.12	0.17	118
Coronary artery spasm	0.78	0.91	0.84	405
Weighted average	0.67	0.73	0.69	523

**Table 2:** Stratified 5-fold cross-validation and independent hold-out test performance of the CNN–LSTM ECG-AI model.

Metric	Mean ± SD	Per-Fold
Accuracy	0.7050 ± 0.0491	0.7512, 0.6555, 0.6388, 0.7578, 0.7218
Precision	0.7750 ± 0.0034	0.7758, 0.7812, 0.7729, 0.7734, 0.7717
Recall	0.8727 ± 0.0912	0.9536, 0.7716, 0.7562, 0.9721, 0.9102
F1	0.8186 ± 0.0405	0.8556, 0.7764, 0.7644, 0.8615, 0.8352
AUC	0.5018 ± 0.0325	0.5190, 0.5239, 0.4678, 0.4585, 0.5399

**Table 3:** Baseline characteristics of patients in the derivation and validation cohorts.

Variable	Derivation (*n* = 1050)		Validation (*n* = 600)		*p* Value
	Available Number	Frequency (%) or Mean ± SD	Available Number	Frequency (%) or Mean ± SD	
Male sex *	1050	522 (49.7)	600	346 (57.7)	0.002
Age, year *	1050	56.3 ± 12.8	600	57.1 ± 11.9	0.190
Smoking *	1050	264 (25.1)	600	199 (33.2)	<0.001
Body mass index, kg/m^2^	1049	26.1 ± 4.3	600	25.8 ± 3.8	0.259
Body surface area, m^2^ *	1049	1.76 ± 0.20	600	1.73 ± 0.19	0.004
Vital sign					
Heart rate, beats/min *	1045	69.4 ± 11.9	593	72.2 ± 13.2	<0.001
Systolic blood pressure, mmHg *	1050	122.5 ± 18.9	597	132.7 ± 21.1	<0.001
Diastolic blood pressure, mmHg *	1050	74.7 ± 10.8	597	77.8 ± 11.9	<0.001
Comorbidity	1050		600		
Diabetes mellitus *		127 (12.1)		116 (19.3)	<0.001
Hypertension *		354 (33.7)		263 (43.8)	<0.001
Left ventricular ejection fraction, % *	1040	65.2 ± 7.3	600	67.2 ± 9.6	<0.001
Laboratory results					
Serum creatinine, mg/dL	1050	0.86 ± 0.32	600	1.04 ± 0.38	<0.001
eGFR, mL/min/1.73 m^2^ *	1050	91.9 ± 23.9	600	76.8 ± 21.6	<0.001
Hemoglobin, g/dL *	1048	13.6 ± 1.6	457	13.5 ± 1.7	0.570
Hematocrit, %	1048	40.0 ± 4.5	457	40.0 ± 4.6	0.932
Total cholesterol, mg/dL *	1046	172.2 ± 37.9	595	203.7 ± 39.4	<0.001
Low-density lipoprotein, mg/dL	1001	101.3 ± 31.9	205	144.9 ± 35.9	<0.001
High-density lipoprotein, mg/dL	1002	46.0 ± 12.7	206	36.4 ± 12.8	<0.001
Platelet counts, 10^9^/L *	1048	230.4 ± 59.3	456	219.5 ± 61.8	0.001
White blood cell count, 10^6^/L *	1048	6843 ± 1752	459	7083 ± 2027	0.020
Monocyte counts, 10^6^/L	1046	495.5 ± 168.2	415	432.1 ± 187.8	<0.001
Coronary artery spasm	1050	709 (67.5)	600	289 (48.2)	<0.001

eGFR, estimated glomerular filtration rate. Data are presented as frequency (percentage) or mean ± standard deviation. * Included in the random forest analysis, yielding final valid sample sizes of 1033 in the derivation cohort and 451 in the validation cohort.

**Table 4:** Baseline characteristics of enrolled patients according to CAS in the derivation and validation cohorts.

Variable	Derivation (*n* = 1050)			Validation (*n* = 600)		
	CAS (*n* = 709)	Non-CAS (*n* = 341)	*p* Value	CAS (*n* = 289)	Non-CAS (*n* = 311)	*p* Value
Male sex	388 (54.7)	134 (39.3)	<0.001	199 (68.9)	147 (47.3)	<0.001
Age, year	57.2 ± 12.1	54.4 ± 14.0	0.001	57.7 ± 12.3	56.6 ± 11.6	0.263
Smoking	204 (28.8)	60 (17.6)	<0.001	127 (43.9)	72 (23.2)	<0.001
Body mass index, kg/m^2^	26.1 ± 4.2	26.0 ± 4.6	0.591	25.8 ± 3.7	25.8 ± 4.0	0.937
Body surface area, m^2^	1.76 ± 0.20	1.74 ± 0.21	0.056	1.74 ± 0.18	1.71 ± 0.19	0.036
Vital sign						
Heart rate, beats/min	68.6 ± 11.7	71.3 ± 12.1	0.001	71.6 ± 12.8	72.8 ± 13.5	0.265
Systolic blood pressure, mm-Hg	120.5 ± 17.8	126.8 ± 20.5	<0.001	130.6 ± 20.3	134.7 ± 21.7	0.017
Diastolic blood pressure, mm-Hg	73.4 ± 10.4	77.3 ± 11.0	<0.001	76.0 ± 11.3	79.5 ± 12.1	<0.001
Comorbidity						
Diabetes mellitus	88 (12.4)	39 (11.4)	0.650	54 (18.7)	62 (19.9)	0.698
Hypertension	246 (34.7)	108 (31.7)	0.332	121 (41.9)	142 (45.7)	0.350
Left ventricular ejection fraction, %	65.6 ± 7.1	64.6 ± 7.7	0.042	66.2 ± 9.1	68.2 ± 9.9	0.012
Laboratory results						
Serum creatinine, mg/dL	0.85 ± 0.26	0.87 ± 0.42	0.448	1.04 ± 0.35	1.04 ± 0.41	0.871
eGFR, mL/min/1.73 m^2^	91.8 ± 22.0	92.0 ± 27.3	0.919	78.1 ± 21.8	75.6 ± 21.4	0.152
Hemoglobin, g/dL	13.7 ± 1.5	13.2 ± 1.6	<0.001	13.8 ± 1.5	13.2 ± 1.8	<0.001
Hematocrit, %	40.5 ± 4.2	38.9 ± 4.9	<0.001	40.7 ± 4.1	39.2 ± 5.0	0.001
Total cholesterol, mg/dL	172.6 ± 37.6	171.4 ± 38.7	0.629	201.7 ± 39.7	205.6 ± 39.0	0.230
Low-density lipoprotein, mg/dL	102.0 ± 31.6	99.7 ± 32.4	0.281	144.3 ± 38.0	145.4 ± 33.7	0.838
High-density lipoprotein, mg/dL	45.4 ± 12.3	47.3 ± 13.3	0.031	35.6 ± 11.7	37.1 ± 13.8	0.415
Platelet counts, 10^9^/L	231.0 ± 59.5	229.0 ± 59.0	0.616	227.0 ± 62.8	211.8 ± 59.9	0.008
White blood cell count, 10^9^/L	6892 ± 1790	6740 ± 1667	0.188	7532 ± 2146	6616 ± 1784	<0.001
Monocyte counts, 10^3^/L	502.9 ± 171.4	479.9 ± 160.7	0.038	495.2 ± 207.0	367.4 ± 139.3	<0.001

CAS, coronary artery spasm; eGFR, estimated glomerular filtration rate. Data are presented as frequency (percentage) or mean ± standard deviation.

**Table 5:** Variable importance of 15 predictors included in the initial random forest model in the derivation cohort.

Feature	VIMP (%)	Rank of VIMP
Diastolic blood pressure	11.38	Top 1
Systolic blood pressure	10.09	Top 2
Age	8.56	Top 3
Body surface area	7.73	Top 4
Hemoglobin	7.39	Top 5
Smoking	5.82	Top 6
Heart rate	5.37	Top 7
Sex	5.34	Top 8
Estimated glomerular filtration rate	4.62	Top 9
Hypertension	1.75	Top 10
Platelet counts	0.29	Top 11
Total cholesterol	0.24	Top 12
Left ventricular ejection fraction	−0.04	Top 13
Diabetes mellitus	−0.77	Top 14
White blood cell count	−1.31	Top 15

VIMP, variable importance.

**Table 6:** The performance of random forest models with different number of predictors according to variable importance in the derivation cohort.

Feature Numbers	AUC, % (95% CI)
All (15 features)	87.8 (85.8 to 89.9)
Top 14 features	87.9 (85.9 to 90.0)
Top 13 features	87.3 (85.2 to 89.4)
Top 12 features	86.3 (84.1 to 88.6)
Top 11 features	86.7 (84.5 to 88.9)
Top 10 features	86.1 (83.8 to 88.3)
Top 9 features *	85.8 (83.6 to 88.1)
Top 8 features	85.2 (82.8 to 87.5)
Top 7 features	85.9 (83.7 to 88.2)
Top 6 features	85.0 (82.7 to 87.3)
Top 5 features	83.4 (80.9 to 85.9)
Top 4 features	82.1 (79.5 to 84.7)
Top 3 features	80.8 (78.2 to 83.5)
Top 2 features	77.1 (74.2 to 80.0)
Top 1 features	65.5 (61.9 to 69.1)

AUC, area under the curve; CI, confidence interval. * The finally selected model.

## Data Availability

The data presented in this study are available on request from the corresponding author due to privacy and ethical restrictions.

## Supplementary Materials

The following supporting information can be downloaded at https://www.mdpi.com/article/10.3390/diagnostics16121847/s1, Table S1. Comparison of binary and categorical cross-entropy performance; Table S2. Comparative performance of CNN–LSTM, K-Nearest Neighbors and Support Vector Machine Models.
