# Supplementary material for: Development and Validation of Artificial Intelligence Prediction of Epicardial Coronary Artery Spasm in Patients Without Obstructive Coronary Artery Disease
Source: Diagnostics (Basel). 2026 Jun 15;16(12):1847. doi: 10.3390/diagnostics16121847 (PMC13298201; doi:10.3390/diagnostics16121847)
Supplement: Supplementary file 1 [file diagnostics-16-01847-s001.zip › diagnostics-4287918-supplementary.pdf]

Supplementary Data

Supplementary Table S1. Comparison of binary and categorical cross-entropy performance.

Item	Binary cross-entropy (Original)	Categorical cross-entropy (New)
Loss function	binary_cross-entropy + sigmoid(1)	categorical_cross-entropy + softmax(2)
Test AUC	0.5365	0.4879
Test accuracy	0.7342	0.6500
Control precision / recall / F1	0.29 / 0.12 / 0.17	0.21 / 0.21 / 0.21
CAS precision / recall / F1	0.78 / 0.91 / 0.84	0.77 / 0.77 / 0.77
Confusion matrix	[[14] [104],[35] [370]]	[[25] [93],[92] [313]]

Supplementary Table S2. Comparative performance of CNN–LSTM, K-Nearest Neighbors and Support Vector Machine Models.

Model	Test AUC	Test Accuracy	Control F1	CAS F1
CNN–LSTM (binary cross-entropy)	0.5365	0.7342	0.17	0.84
CNN–LSTM (categorical cross-entropy)	0.4879	0.6500	0.21	0.77
K-Nearest Neighbors (k=5, PCA-100)	0.5271	0.7200	0.20	0.83
5-fold ensemble (CNN–LSTM)	0.4976	0.7667	0.12	0.87
Support Vector Machine	0.5300	0.7700	0.00	0.87

CAS, coronary artery spasm; CNN–LSTM, convolutional neural network-long short-term memory; PCA, Principal Component Analysis.
